# Return and volatility transmission between oil price shocks and agricultural commodities

**DOI:** 10.1371/journal.pone.0246886

**Published:** 2021-02-19

**Authors:** Zaghum Umar, Mariya Gubareva, Muhammad Naeem, Ayesha Akhter

**Affiliations:** 1 College of Business, Zayed University, Abu Dhabi, United Arab Emirates; 2 South Ural State University, Chelyabinsk, Russian Federation; 3 ISCAL – Lisbon Accounting and Business School Instituto Politécnico de Lisboa, Lisbon, Portugal; 4 SOCIUS / CSG - Research in Social Sciences and Management Rua Miguel Lupi, Lisbon, Portugal; 5 UCP Business School, University of Central Punjab, Lahore, Punjab, Pakistan; Institute for Economic Forecasting, Romanian Academy, ROMANIA

## Abstract

This paper studies the connectedness between oil price shocks and agricultural commodities. Our sample period ranges from January 2002 to July 2020, covering the three global crises; Global Financial Crisis, the European sovereign debt crisis and Covid-19 pandemic crisis. We employ Granger causality tests, and the static and dynamic connectedness spillover index methodology. We find that the shocks in oil prices are Granger-caused mainly by price changes of grains, live cattle, and wheat, while supply shock granger causes variations mostly in grain prices. We find that, from the point of view of static connectedness, for both, price and volatility spillovers, the livestock is the largest transmitter, while the lean hogs are the major receiver. Our dynamic analysis evidences that connectedness increases during the financial crisis period. Our results are potentially useful for investors, portfolios managers and policy makers.

## 1. Introduction

Crude oil is recognized as the world’s most important raw material and essential energy resource since many years. At present, it has become a key element for socio-economic development and stability. Furthermore, petroleum products, such as diesel, gasoline and other fuels, represent important energy source for farming machinery, being consumed by fleets of agricultural machinery and transport vehicles used for the production process in agriculture fields [1, 2]. In this way, growing crude oil prices may result in rising costs of the farming production that, in its turn, may cause a general increase in agricultural commodity prices [2–4]. As per global statistics, a steady growth of agricultural commodities´ prices was observed since 2003 up to 2008, with a phenomenal increase in the upward trend from early 2006 to mid-2008. However, in the second half of 2008 a considerable decline took place in commodity prices driving them back to the levels of early 2007 [5]. Although commodity prices have somewhat recovered before the Covid-19 economic crisis, they were severely affected by the drop in demand due to the pandemic and currently continue being low in comparison to their historical levels as well as to financial assets in general. As risks of inflation due to an unseen expansion of monetary and fiscal policies by the major central banks around the world are potentially threatening global economy and investors, the real assets, as opposite to financial instruments, i.e., commodities, are becoming the assets of interest for hedging eventual spikes in inflation and for portfolio diversification. Thus, the global dynamics of energy and agricultural commodities prices have recently become a serious topic for academy researchers, market practitioners, and policy makers around the globe.

Due to the financialization of commodities, the role of commodities in portfolio diversification and risk management has attracted a lot of attention [6–11]. In particular, a special interest has been attracted by the co-movements of agricultural commodities´ and energy resources´ prices. A renewed interest in the investigation of this relationship started at the end of the first decade of the 21-st century, when a sharp simultaneous increase in crude oil and non-energy commodities´ prices was observed during the last two years, which precede the global financial crisis of 2007–2008. The examination of this co-phenomena can be attributed to three main mechanism underlying the relationship between crude oil and agricultural commodities´ prices. First, during that time, the higher prices of crude oil were found to be responsible for the so-called “food crisis”. It is worth mentioning that as a result of increasing global economic activity, food prices and many agricultural product prices experienced frequent price hikes [4, 12]. Second, another important reason behind this so-called “food-crisis” can be attributed to the substitution effect between fossil fuels and biofuels. Due to this, the higher oil prices cause the demand for biofuels to reach at a higher level. The oil price shocks inspire people to develop alternative energy sources. Among these energy sources, we highlight biodiesel and bioethanol, which are mainly produced from corn and soybean, respectively. These two biofuels are commonly considered as close substitutes for petroleum fuels, such as diesel and gasoline [4, 12]. Ciaian and Kancs [13] document that the rapid increase in production of biofuels during the years 2004–2008 led to a higher degree of co-movement between the prices of the agricultural commodities and fossil fuels. In particular, Ciaian and Kancs [13] found that in the U.S. the post-May-2006 increase in ethanol production led to an increased demand for corn, becoming more closely aligned with prices of energy commodities. Third, an increase in oil prices results in growing production costs of agricultural products, due to the oil-dependent inputs, such as fertilizers, production machinery, and transport. The increases in prices of such inputs are passed through to the agricultural commodities´ prices and made these products expensive. Thus, oil prices can directly lead to higher agricultural commodities´ prices [14–16]. Apart of the three above mentioned mechanisms, there are other factors, such as, e.g., real interest rate [17] and exchange rate [18], which also could partly explain an interdependency existing between crude oil and agricultural commodities´ prices.

In parallel with interrelation between energy and non-energy commodities´ markets, a special attention of scientific community has been being paid to studies of shocks of crude oil prices. For instance, Kilian [19] document the importance of distinguishing between the sources of oil shocks, namely: demand and supply shocks. He studies oil price shocks employing a structural vector autoregressive (SVAR) model, which integrates data for oil production with data for shipping prices, used as proxies for demand and supply shocks, respectively. The findings of this study show that every shock has a different effect on US macro-economic indicators. Kilian´s [19] approach, is commonly employed by a vast body of researchers to investigate diverse impacts of various oil price shocks on systemically important macro-economic parameters.

Kilian [19] SVAR methodology is extensively adopted for the analysis of the impact of oil price shocks on various economics and financial variables. However, Ready [20] points out that this approach has one important shortcoming that the employed data need to be associated with the proper shifts in prices of crude oil to discern whether an analysed shock is primarily due to demand or supply side. Furthermore, it is difficult to find out whether increase in demand is motivated by probability of future variations in demand factor or by expectations relative to supply conditions. E.g., the SVAR methodology is unable to appropriately treat an increase in oil prices due to an augmented probability of a decreasing supply that never comes true. The same is valid for the oil price changes driven by not finally materialized expectations relative to demand side. Ready [20] proposed an alternative technique by distinguishing between demand- versus supply-driven movements in oil prices. This approach is based on the notion that share prices of the companies mostly involved in oil production benefit by an increase in demand for “black gold”, which motivates them to sell larger volumes at potentially higher prices. However, their stock capitalization is rather insensitive to shocks on the supply side, as negative decaying-volume-driven impacts on revenues of these companies, are on the other hand, compensated by higher prices of sales under such conditions. Hence the dynamics of share prices of oil producing companies can be used to distinguish those shocks in prices of crude oil, which are triggered by the concerns regarding the demand side, from those related to worries about supply. Ready [20] empirically documents that the SVAR model, incorporating the above demand-supply distinction, offers a fairly appropriate treatment of both, demand- and supply-driven shocks.

Another distinct advantage of the methodology proposed by Ready [20] is that it allows us to compute daily oil shocks, whereas the Kilian´s [19] approach allows to compute only lower frequency shocks: mostly on quarterly or on monthly basis. That is why Kilian’s [19] approach, being suitable for studies involving macroeconomic aggregates, such as gross domestic product (GDP) and economic growth rate, is not an adequate way to analyse high frequency data such as share price dynamics, commodity prices, exchange rates,. For such financial time series, daily frequency enable us to uncover interesting patterns and therefore is a more desirable choice. Therefore, in this paper we adopt the Ready´s [20] methodology to disentangle oil shocks into demand, risk and supply shocks.

Having disentangled the oil price shocks, we study how they impact prices and volatility of agricultural commodities. Intricate bi-directional interdependencies between agricultural commodity and crude oil prices rather complex communication networks, involving global macroeconomic conjuncture, inflation trends and monetary and fiscal policies. Hence, appropriate tools, capable of capturing complexities of bi-directional causality interactions, are required for tackling such challenges. Therefore, in our paper we use the methodology proposed by Diebold and Yilmaz [21–23] to address the issues of network time-varying connectedness. Thus, the methodology that we follow in our research is fairly robust and allows to account for non-linear price movements of agricultural commodities and crude oil, as well as for bi-directional influences between them. This framework is extensively used in financial time series analysis including equities, fixed income securities, exchange rates, commodity markets [24]. In our methodology, the connectedness appears not just as a result of a mutual reliance of SVAR variables on one another; in parallel we account for interdependencies of shocks, observed in prices of crude oil and agricultural commodities. We also examine how connectedness varies along the time by selection rolling window sample. Thus, we try to extend he literature on the relationship between oil prices and agricultural commodities by employing novel financial econometric techniques that enable us to uncover the relationship between three oil specific shocks and the agricultural commodities at a higher frequency. To the best of our knowledge, this is the first study to analyse the oil-agricultural market relationship by combining these two unique methodologies. Having in mind that the demand, supply and risk factors are among the major drivers for oil prices shocks, our motivation is to study the influence of these three factors on the interdependence between agricultural commodity and crude oil prices, as well as on the interrelations of their volatilities in the context of US economy. We use a novel econometric approach for the achievement of this fundamental research objective. Our sample data cover 11 agricultural commodity aggregates of the US market, namely, wheat, cocoa, soybeans, sugar, cotton, grains, coffee, live cattle, feeder cattle, lean hogs and livestock from the time period of 2002 to 2020. Our results from granger causality analysis show that oil price shocks have a causal relationship with price changes of grains, live cattle, and wheat. Furthermore, our return and volatility connectedness analysis show that the livestock is the largest transmitter, while the lean hogs are the major receiver of shocks in the system of oil price shocks and the agricultural commodities. We show that the connectedness of the oil-agricultural commodities increases during financial crises.

The rest of the paper is structured as follows. In Section 2 we present a chosen literature, relevant to our research. Section 3 is dedicated to the methodology. Section 4 describes data and sample statistics. Section 5 presents empirical results and the implications. Section 6 concludes.

## 2. Literature review

A vast body of researchers have been exploring the relationship between shocks in crude oil prices and movements in prices of agricultural commodities, as potentially any variation in oil price can affect the whole economy. We start our literature survey by having a closer look at the empirical studies of famous scholars which could help uncovering the interrelationship between price dynamics of energy and non-energy commodities.

The seminal work by Esmaeili and Shokooni [25] provides theoretical reasoning of the linkage between oil and agricultural commodity prices, which currently an important concern for any economy. In particular, their study explains that fluctuations in prices of crude oil can significantly impact the socio-economic activities all around the world. They also conclude that oil prices can affect the world’s GDP due to their influence on food production costs. Further on, Zhang and Qu [26] investigate how prices of agricultural commodities in China are impacted by global shocks in prices of crude oil. They examine an extensive set of agricultural commodities and find that oil shocks on majority of them are asymmetric.

Another paper by Koirala et al. [27] focuses on correlation between agricultural commodities and crude oil returns. According to their findings, 15 billion gallons of corn ethanol have been produced in the US in 2015 under the US energy policy acts and regulations. They also find that prices of energy and non-energy commodities exhibit high degree of correlation. This finding points out that the energy-intensive agriculture plays an intermediary role in creating a linkage between energy and agricultural sector. The authors´ conclusion is that increasing energy prices can make prices of agricultural commodities grow. In a similar effort, Fowowe [28] investigates the same relationship in the context of South Africa. He reports that structural break cointegration does not exhibit long-term connection between prices on energy resources and those of food products and that the nonlinear causality tests also evidence the absence of short-term relationship between food and energy prices. His results indicate that prices of agricultural products in South Africa are insensitive to the prices of crude oil.

However, in a more recent paper by Pal and Mitra [29], focused on the global economy, it is evidenced that the food-fuel interrelations have sharply strengthened after the global financial crisis. In addition, the authors perform cross-correlation analyses and conclude for existence of positive and strong interrelations between prices of agricultural and energy commodities.

In respect to the subjacent methodologies employed to study food-fuel nexus, we mention the study Wang et al. [30]. Employing structural VAR models, the authors investigate the price dynamics of energy resources and food products. This study finds that oil shocks explain from 20% to 40% of changes in prices of agricultural commodities. However, the impacts of these shocks have become more pronounced after the global financial crisis (GFC). During the pre-crisis period, oil shocks explain only a minor share of changes in the price of food products, while their influence become more evident after the GFC.

As a next important study we discuss Locutte [31], who uses bivariate vector autoregression (VAR) models to investigate co-movements between food and crude oil prices. He also empirically evidences an existing linkage between energy and non-energy commodities prices. The author finds a notable distinction between the pre-commodity-boom window and the most recent post-crisis history, stating that differently from the former time interval, during the latter strong positive co-movements are observed and documented.

A more recent study of Adam et al. [32] examines causality issues relationship between three following variables: the global price of oil, the IDR/EUR foreign exchange rate and the price of Indonesian rice. The authors use historical time series on a monthly basis covering the period of 2000–2017. They employ VAR methodology and find that there is no relationship in the long run between the three above-mentioned parameters. A meaningful relationship was only detected in a short run. The results of Granger causality test evidence that the direction of this relationship is from both, the IDR/EUR exchange rate and the price of crude oil toward the price of rice.

It is worth noting that the study of Ready [20], previously mentioned in the Introduction, explains an advanced innovative methodology, based on share price behaviour of oil producing corporations, which allows to classify changes in crude oil prices either as supply-driven or as originated at the demand side. The author shows that economic outputs and, thus, equity returns of these stocks are strongly correlated with demand shocks, whereas supply-driven price movements are proved to be hardly noticeable in the respective stock price dynamics. Malik and Umar [33] employ this methodology to disentangle different types of shocks in crude oil prices using daily frequency time series. They analyse the influence of oil-supply-driven and oil-demand-driven shocks on foreign exchange rates of major oil-dependent economies. In line with Ready [20], their empirical findings evidence that demand-driven shocks exercise a stronger influence over exchange rate dynamics while supply-driven shocks produces only very limited effects. The interconnectedness between oil price movements, on one hand, and exchange rates, on the other hand, is found to considerably strengthen after the GFC. These important outcomes are potentially useful for academy researchers, market practitioners, and regulators.

Another strand in the literature addresses the relationship of volatility spillovers between crude oil and agricultural commodities prices. Beckmann and Czudaj [34] examined the volatility of corn, cotton, and wheat futures and the volatility spillovers across these markets. This study is based on the GARCH-in-mean vector autoregressive models. The results evidenced a short-run volatility transmission process. Later, Ahmadi et al. [35] explore the influence of shocks in crude oil prices shocks on the price volatility of agricultural commodities. Their empirical analysis is based on the structural VAR methodology and employs impulse response functions. The authors find that volatilities in commodity prices react differently to various oil shocks. They also report that during the GFC, the impacts produced by oil shocks are generally stronger than during the pre-crisis and post-crisis periods.

Lu et al. [36] examines dynamics of spillovers between crude oil and agricultural commodities prices for the period beginning with the aftermath of the GFC. For this purpose, the authors adopt a bivariate VAR methodology. They conclude that, in a short run, the spillovers are bidirectional whereas in a middle and long run volatilities of corn are passed through to the volatility of oil prices. Lu et al. [36] conclude that energy and non-energy commodities after the GFC present indications of lower degree of integration.

On the other hand, it is worth commenting on research in network connectedness. In this context we mention the seminal paper by Diebold and Yilmaz [23] who analysed diverse metrics of connectedness, addressed by decomposing the respective variances of the considered time series. The variance decompositions, which define directed networks with different weights assigned to network links similarly to the approach widely spread in the network literature, serve as a base for the method providing intuitive and comprehensive connectedness metrics. From the applied perspective, Diebold and Yilmaz [23] document the day-to-day connectedness of the stock return volatility of major US financial institutions.

Despite there are an emerging research activity on the interdependence between shocks in crude oil prices, agricultural commodity returns, and volatility, to the best of our knowledge there does not exist in the literature any paper, employing Ready´s [20] methodology allowing for daily frequency time series, that addresses the influence of isolated shocks in oil prices on the returns and volatility of agricultural commodities. Our paper fills this void.

## 3. Methodology

We follow a two-step methodology. First, we segregate the oil price shocks into the three components: demand-driven, supply-driven, and those related to changes in a risk perception by employing the Ready [20] framework based on share price dynamics of major oil producing companies. Following Ready [20], we employ the DataStream World Integrated Oil and Gas Producer Index as a proxy for oil producing firms. Similarly, in order to account for oil price changes, we employ the crude oil futures’ returns with a maturity of one month listed on the New York Mercantile Exchange. Lastly, we use VIX index as measure of risk perception. Following Ready [20], we identify the unexpected changes in VIX by employing a ARMA (1,1) process. The demand shocks are decomposed from the return component of the oil producing firms index that is independent of unexpected changes in VIX. Supply shocks are the component of the oil price changes that are independent of the other two (demand and risk) shocks. Ready [20] empirically documents the appropriateness of the shocks, constructed by employing this approach, in accounting for all possible oil price changes.

In order to decompose the variances of the 14-variables system, encompassing the shocks in prices of oil and agricultural commodities, we follow Diebold and Yilmaz [23] methodology, as it allows broadly defining the system networks. The technique can be given as follows.: First, we fit a vector autoregressive (VAR) model to the system of oil price shocks and the commodities; second, using the data to time t, we estimate a forecast based on available data up to the time t+H; third, for each component, we decompose the error variance of the forecast for the shocks attributed to each component in the system at time t. This technique is closely related to general methods of variance decomposition commonly used in econometrics.

To model the connectedness, let us use $d_{ij}^{H}$ to represent the H-step variance decomposition component of variable i due to shocks in variable j. The connectedness measures use are based on ‘‘non-own”, or ‘‘cross”, variance decompositions, $d_{ij}^{H}$, i,j, = 1,…, N, i≠j.

Consider an N-dimensional covariance-stationary data-generating process (DGP) with orthogonal shocks: *x*_*t*_ = Θ(*L*)*u*_*t*_, Θ(L) = Θ0 + Θ_1_*L* + Θ_2_*L*^2^ + …, *E*(*u*_*t*_*u*_*t*_′) = *I*. Note that Θ_0_ need not be diagonal. All aspects of connectedness are contained in this general representation. Contemporaneous aspects of connectedness are summarized in Θ_0_, and dynamic aspects in {Θ_1_, Θ_2_, …}. However, the information in these estimated coefficients is typically esoteric and we need to transform this information into a more comprehendible but compact measure of connectedness. We transform these coefficients using variance decompositions. Let’s denote the “variance decomposition matrix” by $D^{H}=[d_{ij}^{H}]$. The off-diagonal entries of D^H^ are the parts of the N forecast-error variance decompositions of relevance from a connectedness perspective, and, they measure the pairwise directional connectedness. The gross pair-wise directional connectedness, in particular, from j to i is given as follows:

$$C_{i\leftarrow j}^{H}=d_{ij}^{H}.$$ (1)

Generally, $C_{i\leftarrow j}^{H}\neq C_{j\leftarrow i}^{H}$, hence, we define the net pair-wise directional connectedness as follows:

$$C_{ij}^{H}\neq C_{j\leftarrow i}^{H}-C_{i\leftarrow j}^{H}.$$ (2)

The off-diagonal row sums provide the share of the H-step forecast-error variance of factor xi received from the shocks arising in other factors, while the off-diagonal column sums give the share of the H-step forecast-error variance of factor xi transmitted to shocks in other factors. Resultantly, the off-diagonal row and column sums, labelled “from” and “to” in the connectedness table, offer the measure of total directional connectedness in the components of the system. The total connectedness from others to i is given as:

$$C_{i\leftarrow •}^{H}=\sum_{\begin{array}{c} j=1\\ j\neq i \end{array}}^{N}d_{ij}^{H},$$ (3)

and total connectedness from j to others is given as:

$$C_{•\leftarrow j}^{H}=\sum_{\begin{array}{c} i=1\\ j\neq i \end{array}}^{N}d_{ji}^{H}.$$ (4)

Similarly, net total directional connectedness is defined as

$$C_{i}^{H}=C_{•\leftarrow i}^{H}-C_{i\leftarrow •}^{H}.$$ (5)

Since the variance of a weighted sum is not an appropriate sum of variances, the variance decompositions for the non-orthogonal shocks are not as easily estimated as discussed above. In this context, traditional techniques like Cholesky-factor identification for providing orthogonal innovations may be sensitive to ordering. Therefore, Koop et al. [37] and Pesaran and Shin [38] proposed a generalized VAR decomposition (GVD) that is invariant to ordering and following Diebold and Yılmaz [23], we prefer to use their technique. The H-step generalized variance decomposition matrix is given as ${\text{D}}^{\text{gH}}\;=\;[d_{ij}^{gH}]$, and $d_{ij}^{gH}$ is:

$$d_{ij}^{gH}=\frac{\sigma_{jj}^{-1}\sum_{h=0}^{H-1}{\left(e_{i}^{\text{'}}\Theta_{h}\sum e_{j}\right)}^{2}}{\sum_{h=0}^{H-1}(e_{i}^{\text{'}}\Theta_{h}\sum \Theta_{h}^{\text{'}}e_{j})},$$ (6)

Where, e_j_ is a selection vector with j^th^ element unity and zeros everywhere, Θ_h_ is the coefficient matrix multiplying the h-lagged shock vector in the infinite moving-average representation of the non-orthogonalized VAR, ∑ is the covariance matrix of the shock vector in the non-orthogonalized VAR, and σ_jj_ is the j^th^ diagonal element of ∑. Sums of forecast error variance contributions are not necessarily unity (that is, row sums of D^g^ are not necessarily unity) as shocks are not necessarily orthogonal in the GVD environment. Hence, we base our generalized connectedness indexes not on Dg, but rather on ${\tilde{D}}^{g}=[{\tilde{d}}_{ij}^{g}]$, where ${\tilde{d}}_{ij}^{g}=\frac{d_{ij}^{g}}{\sum_{j=1}^{N}d_{ij}^{g}}$. By construction $\sum_{j=1}^{N}{\tilde{d}}_{ij}^{g}=1$ and $\sum_{i,j=1}^{N}{\tilde{d}}_{ij}^{g}=N$.

## 4. Data

The sample data consists of the S&P GSCI indices for eleven agricultural commodities, namely, wheat, cocoa, soybeans, sugar, cotton, grains, coffee, live cattle, feeder cattle, lean hogs, and livestock. We use them to study the connectedness between shocks in prices and price volatilities of crude oil and agricultural commodities. We use high frequency daily based data from January 2002 to July 2020 obtained from DataStream. Table 1 below presents basic descriptive sample statistics for each series used in the present research. As per Table 1 Jarque-Bera test results for all commodities as well as high kurtosis observed in all series indicate that the series are non-normal.

**Table 1:** Descriptive statistics of agriculture commodities time series.

	Cocoa	Coffee	Cotton	Feeder cattle	Grains	Lean hogs	Live cattle	Livestock	Soybeans	Sugar	Wheat
Mean	0.01%	0.01%	0.01%	0.01%	0.01%	0.00%	0.01%	0.01%	0.01%	0.01%	0.01%
Maximum	8.99%	12.06%	6.94%	7.48%	7.69%	9.81%	5.45%	5.30%	6.43%	8.56%	15.60%
Minimum	-10.0%	-11.2%	-7.1%	-6.0%	-8.6%	-12.5%	-6.4%	-6.2%	-7.3%	-12.4%	-9.8%
Std. Dev.	1.8%	2.0%	1.6%	1.0%	1.5%	1.8%	1.0%	1.0%	1.5%	2.0%	1.9%
Skewness	-26.9%	11.3%	-6.8%	-10.7%	1.8%	-3.1%	-19.2%	-20.7%	-22.7%	-20.8%	21.0%
Kurtosis	556.6%	498.2%	453.2%	569.3%	534.1%	516.3%	532.2%	505.4%	545.3%	512.0%	578.7%
Jarque-Bera	1384.20	801.30	476.19	1468.88	1103.71	942.65	1114.86	883.68	1252.74	939.24	1598.45
Probability	0.00	0.00	0.00	0.00	0.00	0.00	0.00	0.00	0.00	0.00	0.00
Sum	0.44	0.70	0.50	0.51	0.54	-0.10	0.39	0.27	0.71	0.38	0.54
Sum Sq. Dev.	1.56	1.86	1.31	0.51	1.04	1.59	0.51	0.47	1.05	1.87	1.68

Note: This table shows the descriptive statistics of the S&P GSCI return series employed. The sample covers the period from January 10, 2002 to July 17, 2020.

## 5. Empirical results

In the next three subsections, we present our findings in respect to Granger causality, static connectedness, and dynamic rolling connectedness between different type of oil price shocks, agricultural commodity returns and volatility for the period of 2002–2020. We explain our empirical results and discuss their implications. The first subsection provides the outcomes of Granger [39] causality test that provides insights regarding the causality relations between energy and non-energy commodities. The second subsection thoroughly discusses the static connectedness between crude oil and agricultural commodity markets. The third subsection explores the dynamic rolling window approach in order to track total time-varying connectedness of the entire system.

### 5.1. Granger causality tests

**Fig 1:**
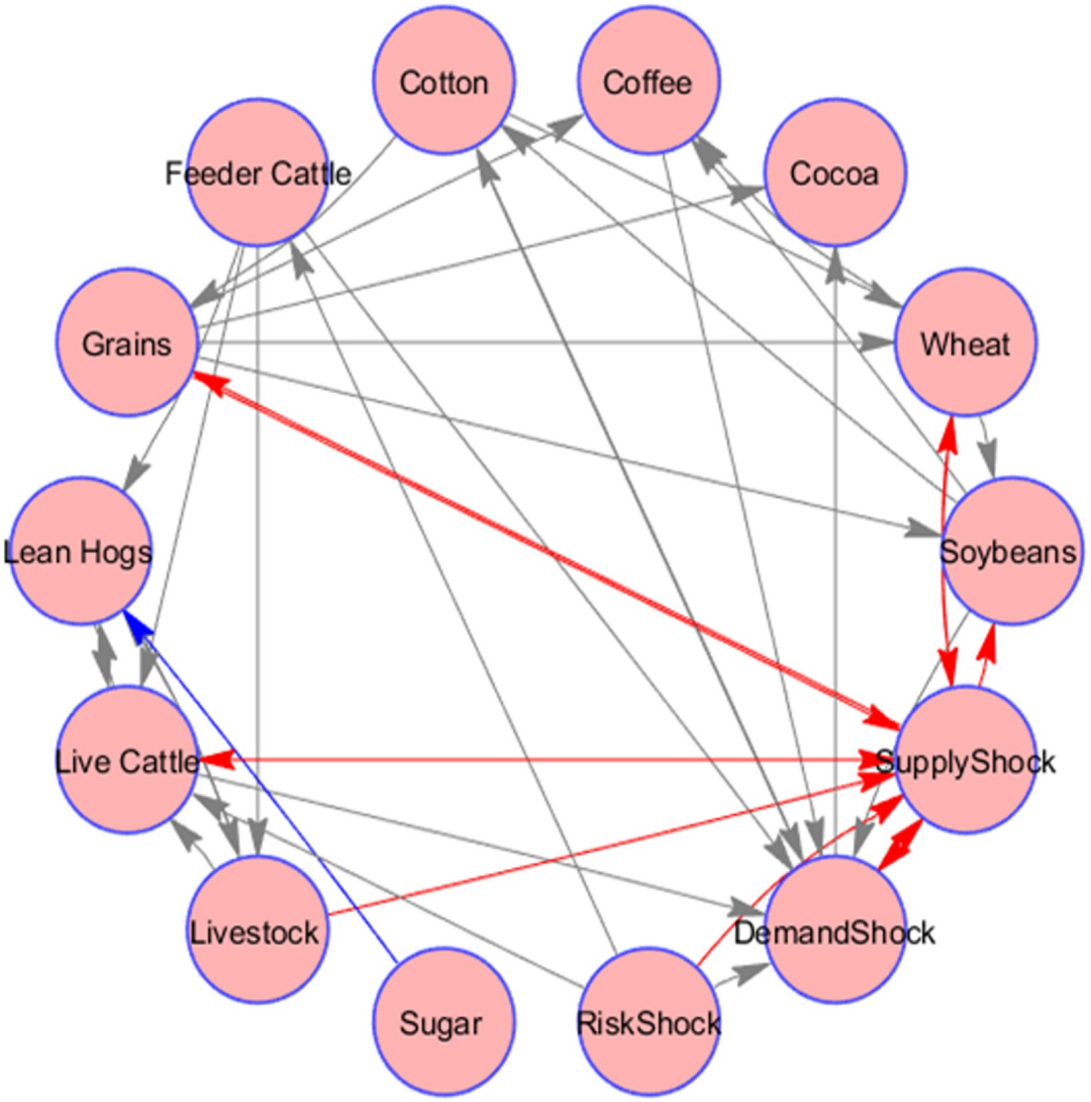
Granger causality test. Fig 1 shows the granger causality results among various agricultural commodities and oil price shocks. Each arrow shows statically significant granger causality (from source to edge of arrow) at 5%.

In the previous literature, there exist many studies that highlight the significance of granger causality tests in explaining the connectedness between various variables. Bearing this in mind, we use the Granger causality test to understand the connection between different oil price shocks and returns of agricultural commodities. In Fig 1, we graphically present the results of causality relationships in the Granger [39] sense.

As per Fig 1, above, the directional arrows indicate the presence of significant causality between oil price shocks and agricultural commodity price movements. It is worth noticing that the risk shocks in oil prices do not cause, in the Granger sense, notable changes in agricultural commodities, and vice versa, non-energy commodity prices do not Granger-trigger abrupt variations of crude oil prices. However, demand-driven and supply-driven shocks do exhibit Granger causality relations of both, out-going and coming-in influences with major part of the considered commodities.

### 5.2. Static connectedness

The whole sample data, compiled in Table 2, represent the average static connectedness measures.

**Table 2:** Static connectedness to agricultural commodities returns and oil price shocks.

	**Soybeans**	**Wheat**	**Cocoa**	**Coffee**	**Cotton**	**Feeder Cattle**	**Grains**	**Lean Hogs**	**Live Cattle**	**Livestock**	**Sugar**	**Risk Shock**	**Demand Shock**	**Supply Shock**	**FROM**
**Soybeans**	48.94	10.37	0.76	1.83	4.13	0.03	26.01	0.4	0.74	0.75	2.09	0.91	2.26	0.79	51.06
**Wheat**	9.69	45.69	0.54	1.47	2.25	0.06	35.36	0.26	0.48	0.48	1.64	0.46	1.05	0.57	54.31
**Cocoa**	1.45	1.01	84.06	2.66	1.44	0.22	1.55	0.16	0.5	0.48	2.23	1.08	2.89	0.27	15.94
**Coffee**	2.97	2.56	2.36	76.07	2.03	0.39	3.76	0.32	0.81	0.9	4.31	0.78	2.33	0.42	23.93
**Cotton**	6.04	3.61	1.11	1.97	72.25	0.14	6.09	0.24	0.5	0.54	2.33	1.94	2.76	0.47	27.75
**Feeder Cattle**	0.01	0.05	0.12	0.18	0.09	44.07	0.09	2.76	27.32	23.97	0.11	0.8	0.43	0.01	55.93
**Grains**	20.21	29.43	0.62	1.8	3.21	0.09	38.02	0.32	0.67	0.61	2.07	0.66	1.58	0.71	61.98
**Lean Hogs**	0.44	0.31	0.1	0.24	0.17	3.64	0.46	59.09	3.97	30.92	0.18	0.25	0.21	0.01	40.91
**Live Cattle**	0.6	0.41	0.23	0.41	0.27	24.87	0.69	2.73	40.03	27.84	0.38	0.8	0.6	0.13	59.97
**Livestock**	0.52	0.35	0.19	0.39	0.25	18.94	0.54	18.45	24.23	34.71	0.29	0.66	0.44	0.06	65.29
**Sugar**	3.29	2.69	1.94	4.28	2.44	0.19	4.11	0.18	0.67	0.59	75.29	0.58	2.63	1.13	24.71
**Risk Shock**	1.55	0.81	1.13	0.85	2.21	1.41	1.44	0.4	1.46	1.44	0.68	86.53	0.06	0.03	13.47
**Demand Shock**	3.66	1.81	2.52	2.64	3.04	0.79	3.31	0.18	1.16	0.91	2.65	1.66	75.41	0.25	24.59
**Supply Shock**	1.35	1.01	0.32	0.46	0.63	0.13	1.67	0.11	0.46	0.39	1.39	0.31	0.45	91.33	8.67
**TO**	51.78	54.43	11.92	19.16	22.17	50.89	85.08	26.5	62.97	89.82	20.34	10.89	17.71	4.86	**TCI**
**NDC**	0.71	0.12	-4.01	-4.77	-5.59	-5.03	23.1	-14.41	3	24.52	-4.38	-2.58	-6.88	-3.81	**37.75**

This table shows the connectedness of the eleven commodity indices and the three oil price shocks. NDC denotes Net directional connectedness and TCI (right bottom corner) denotes the total connectedness index.

Let us now explain the information conveyed by Table 2. The right bottom corner of Table 2 shows the total connectedness index (TCI) of the system, which shows a moderate value of 37.75%. Lets now look at the constituents of the total connectedness index. The off-diagonal numbers exhibit the pairwise connectedness of return spillovers across different markets. In contrast, the on-diagonal numbers indicate within-market return spillovers. For all the types of agricultural commodities, one could clearly see that the connectedness of the commodity returns to crude oil shocks is rather bidirectional than unidirectional. The total connectedness of the entire system is fragmented into two forms. First, the row, denominated ‘TO’, measures the transmission of shocks from each agricultural commodity and three shocks in oil prices towards the entire system(all other variables). This row is calculated as the sum of all the vertical numbers excluding the on-diagonal numbers. Second, the columns, denominated ‘FROM, expresses the aggregate amount of shocks, which all the considered agricultural commodities and the three oil shocks are receiving from the entire system. We estimate the value of this column by adding all the horizontal numbers with the exclusion of on-diagonal numbers.

In particular, the three right-most cells of ‘TO’ row quantify the influence of the risk-, demand-, and supply-driven shocks in oil prices, respectively, transmitted to the remaining body of the system, i.e. the entire system without the respective source of spillover. We notice that the largest contributor of ‘to’ spillover is Livestock (89.82%) followed by Grains (85.08%). The connectedness of oil risk, demand, and supply shocks is 10.89%, 17.71%, and 4.86%, respectively. Thus, supply shocks contribute the lowest to the system. The last column of Table 2 shows the spillover from the system to each variable. Here again, we see that Livestock(65.29%) and grains (61.98%) appear to be the highest recipient of the spillover from the system. In order to distinguish between the net transmitters and net receivers of the spillover, we look at the last row of Table 2, which depicts the net directional spillover. Livestock and Grains ate the highest net transmitters of spillover. In addition, live cattle, soyabean and wheat also are net transmitters. All other variables in the system including the three oil price shocks are net receivers of spillover. Among the agricultural commodities, lean hogs are the significant shock recipient with the net directional connectedness of -14.41%. Livestock is found to be the most relevant net transmitter of inter-variable shocks having 24.52% net directional connectedness.

**Fig 2:**
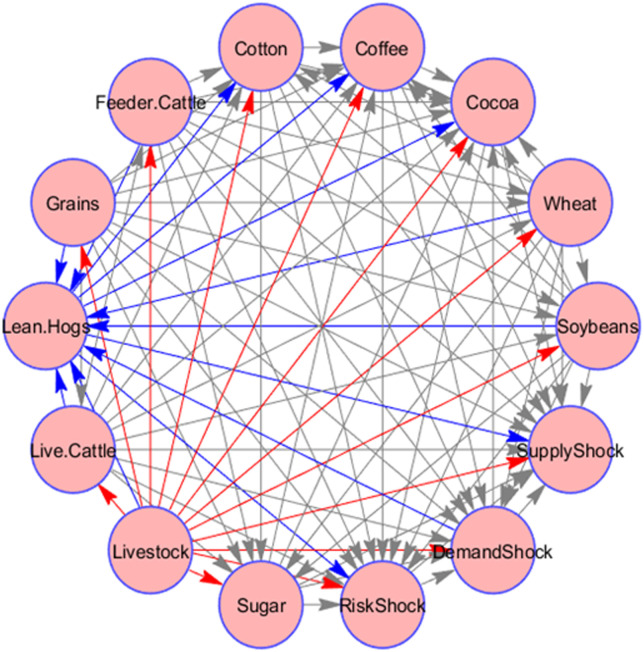
Network graph of static connectedness of commodity returns and oil shocks. This figure shows the pairwise connectedness between agricultural commodity returns and oil shocks. The source of arrow shows the transmitter of spillover and the edge of arrow shows the recipient of the spillover.

Fig 2 presents a help of a network connections graph for the considered system consisting of 14 nodes-variables. The directed arrows indicate the pairwise directions of the static connectedness, coinciding with the flow of bivariate between-nodal influence transmission. In accordance with Table 2, in Fig 2 the most relevant transmitter, the livestock category, is put in evidence by the red-coloured out-going arrows, while the major receiver, Lean Hogs node, is highlighted by the blue colour. In respect to the latter node, it is comprehensible that it possesses a connectedness profile different from those of agricultural commodities but similar to price shocks of crude oils, as Lean Hog is a type of pork futures contract. This feature places it rather within financial than agricultural markets. In fact, we see that Lean hogs are much more sensitive to external influence than other agricultural commodities, and from this perspective is somewhat similar to the shocks in global information-dependent oil prices. As per Fig 2, we can also conclude that, in our network, oil shocks are net recipient of systemic shocks form agricultural commodities.

**Fig 3:**
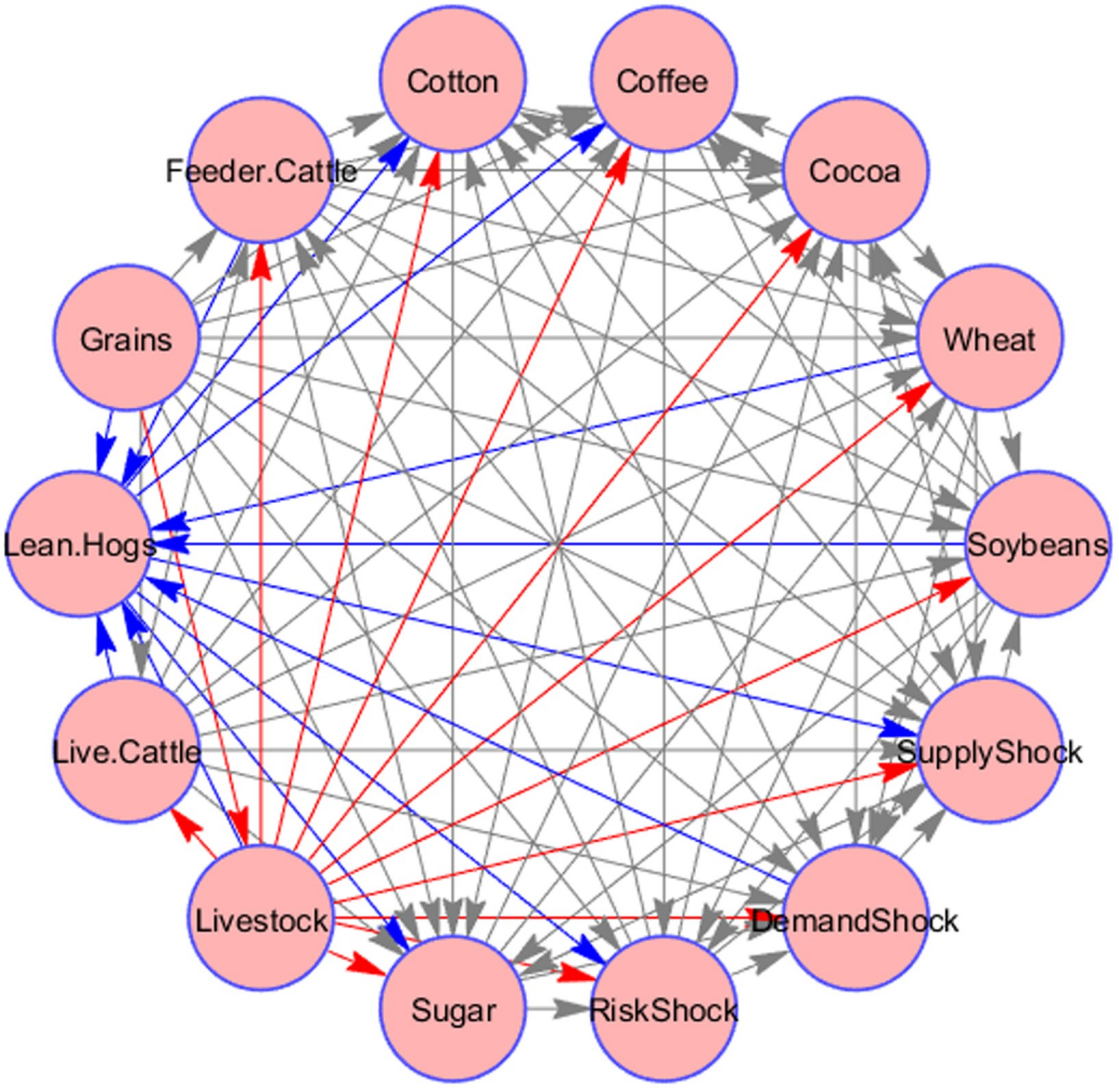
Network graph of static connectedness of commodity volatility and oil shocks. This figure shows the pairwise connectedness between agricultural commodity volatility and oil shocks. The source of arrow shows the transmitter of spillover and the edge of arrow shows the recipient of the spillover.

Table 3 presents the connectedness results for the system encompassing oil shocks and agricultural commodity volatilities. Similar to the return connectedness, we note that livestock and grains appear to be the main transmitter and receiver of shocks from the system. However, when we look at the net directional connectedness figure, we notice that risk shocks from oil appear to be net transmitters of shocks along with Livestock, grains, and Live Cattle. Fig 3 shows the pairwise connectedness of the volatility of commodity indices and the oil price shocks. We can see that Livestock exhibits the highest pairwise transmitter of spillover, whereas, lean hogs represents the highest pairwise recipient of spillover.

**Table 3:** Static connectedness to agricultural commodities volatility and oil price shocks.

	**Soybeans**	**Wheat**	**Cocoa**	**Coffee**	**Cotton**	**Feeder Cattle**	**Grains**	**Lean Hogs**	**Live Cattle**	**Livestock**	**Sugar**	**Risk Shock**	**Demand Shock**	**Supply Shock**	**FROM**
**Soybeans**	65.22	5.19	0.43	0.51	2.47	0.64	23.38	0.44	0.34	0.52	0.65	0.14	0.03	0.05	34.78
**Wheat**	4.64	58.52	0.2	0.3	0.63	0.41	34.21	0.18	0.27	0.15	0.28	0.1	0.06	0.05	41.48
**Cocoa**	0.65	0.18	95.72	0.7	0.23	0.19	0.44	0.11	0.31	0.15	0.59	0.34	0.1	0.28	4.28
**Coffee**	0.8	0.35	0.71	94.47	0.34	0.06	0.47	0.07	0.2	0.37	1.71	0.17	0.17	0.1	5.53
**Cotton**	3.1	0.76	0.21	0.34	90.84	0.13	1.96	0.44	0.34	0.6	0.54	0.56	0.01	0.16	9.16
**Feeder.Cattle**	0.63	0.4	0.14	0.05	0.08	61.13	0.89	0.39	20.68	15.3	0.1	0.11	0.04	0.07	38.87
**Grains**	17.59	28.97	0.21	0.29	1.19	0.67	49.64	0.28	0.24	0.29	0.45	0.1	0.02	0.06	50.36
**Lean.Hogs**	0.75	0.21	0.11	0.04	0.25	0.52	0.67	75.59	1.05	20.39	0.21	0.02	0.13	0.04	24.41
**Live.Cattle**	0.22	0.19	0.16	0.11	0.18	17.93	0.27	0.84	53.9	25.82	0.1	0.1	0.06	0.12	46.1
**Livestock**	0.47	0.07	0.05	0.13	0.3	12.29	0.3	13.52	23.58	49.03	0.16	0.02	0.04	0.06	50.97
**Sugar**	0.94	0.22	0.57	1.76	0.75	0.14	0.61	0.26	0.17	0.31	93.68	0.29	0.09	0.2	6.32
**Risk Shock**	0.16	0.16	0.27	0.38	0.5	0.09	0.15	0.09	0.1	0.04	0.32	97.63	0.07	0.04	2.37
**Demand Shock**	0.04	0.1	0.15	0.17	0.05	0.02	0.04	0.06	0.13	0.14	0.27	2.13	96.38	0.32	3.62
**Supply Shock**	0.04	0.14	0.15	0.18	0.07	0.17	0.21	0.06	0.19	0.17	0.06	0.35	0.53	97.68	2.32
**TO**	30.03	36.93	3.36	4.96	7.03	33.27	63.6	16.74	47.59	64.25	5.46	4.43	1.36	1.55	**TCI**
**NDC**	-4.75	-4.54	-0.92	-0.56	-2.13	-5.6	13.24	-7.67	1.49	13.28	-0.86	2.06	-2.26	-0.77	**22.9**

This table shows the connectedness of volatility of the eleven commodity indices and the three oil price shocks. NDC denotes Net directional connectedness and TCI (right bottom corner) denotes the total connectedness index.

### 5.3. Dynamic rolling connectedness

**Fig 4:**
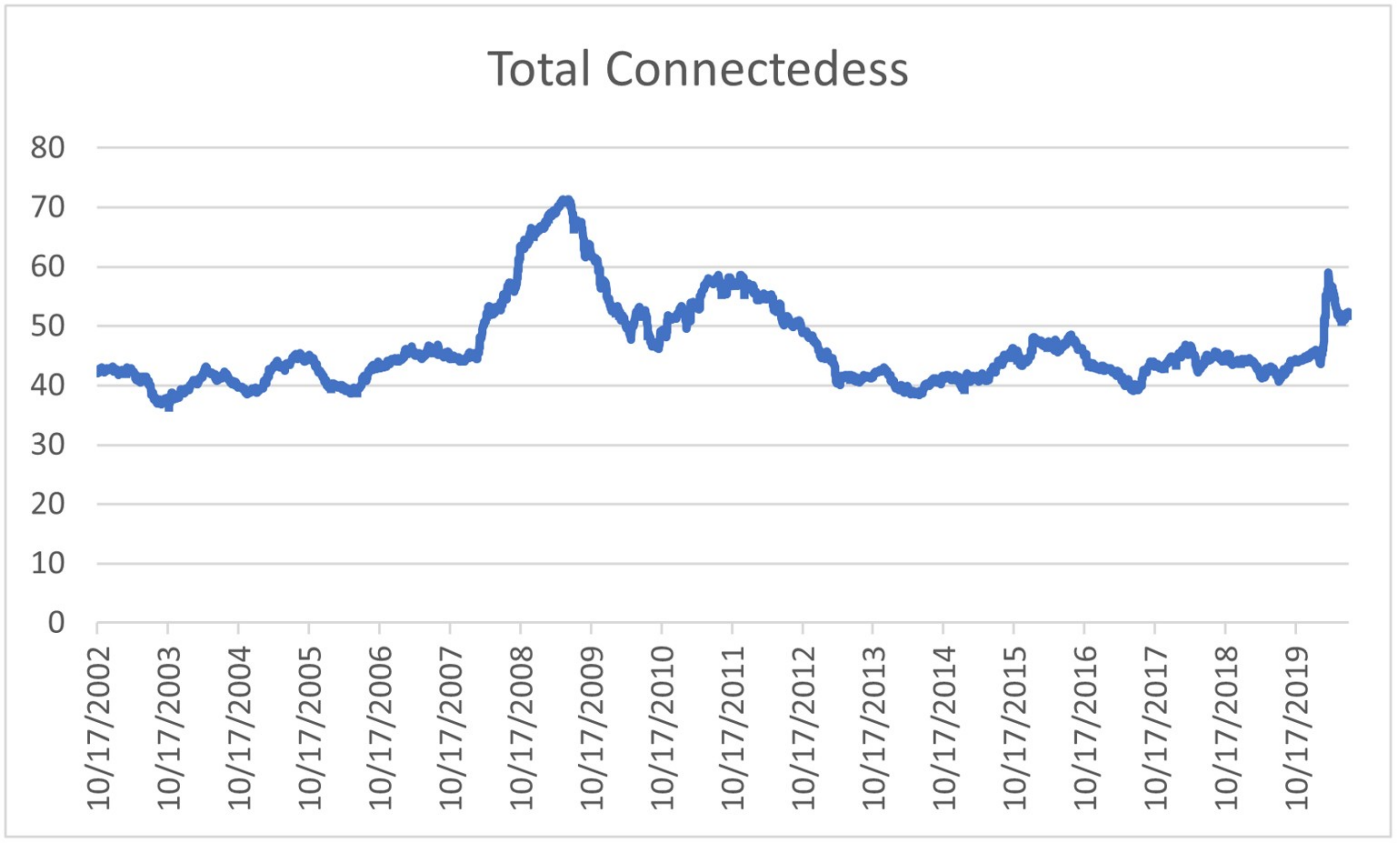
Dynamic Total connectedness of commodity returns and oil price shocks. This figure shows the dynamic connectedness of the commodity returns and oil price shocks by using a following window of 200 days.

In this subsection, we discuss the time dynamics of interdependencies across our commodities-plus-oil system by means of assessing the dynamic connectedness. For this purpose, instead of the entire observation interval, we use a rolling window of 200 observations (approximately 9 months of data). In Fig 4, we graphically illustrate how the total connectedness of the commodity returns and oil price shocks, varies within the period of our study due to the advancement of the rolling window. As per Fig 4, we observe that that the behaviour of the total connectedness exhibits a peak during the global financial crisis (GFC), followed by a relatively lower peak around the European sovereign debt crisis(ESDC) and another spike around the end of the sample period due to the Covi-19 pandemic. The increased connectedness of crude oil and agricultural commodities prices indicates that under conditions of financial stress, the diversification attributes of energy and non-energy commodities seemingly diminish as they all begin to behave in consonance with the stressful influence external to the commodities-plus-oil system.

**Fig 5:**
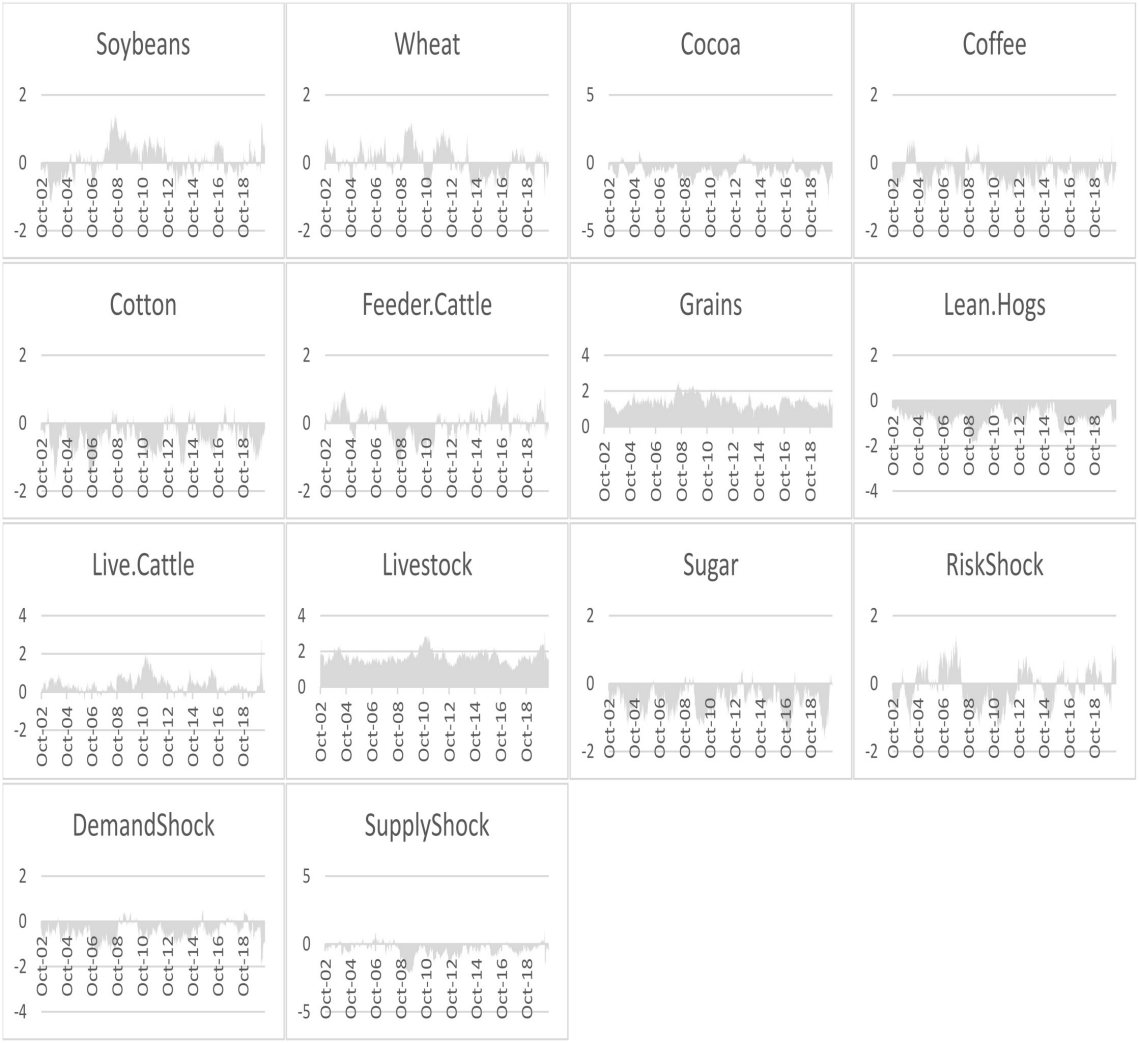
The net directional connectedness of each variable. This figure shows the net directional connectedness the commodity returns and oil price shocks.

In order to see the net transmitters and net recovers of spillover over the sample period, we present the dynamic net directional connectedness of each variable in Fig 5. We note that Grains, Livestock, Live cattle are net transmitters during the entire sample period, whereas, Lean Hogs and Sugar appear to be net receivers of spillover during the entire sample period. Interestingly, Soyabeans and wheat appear to be net transmitters during the GFC and ESDC. As for the oil shocks, we see that the risk and the supply shock were net recivers during the GFC and ESDC, however, the demand shock was a net transmitter during the GFC and net receiver during the ESDC. During the Covid-19 episode, we notice that risk shock is a transmitter, whereas demand and supply shocks are net receivers.

## 6. Conclusion

This paper studies how demand-, supply-, and risk driven changes in crude oil prices are connected to the prices of agricultural commodities. The sample data consists of price for eleven agricultural commodities, namely, wheat, cocoa, soybeans, sugar, cotton, grains, coffee, live cattle, feeder cattle, lean hogs, and livestock. We use them to study the connectedness between shocks in prices and price volatilities. We employ Ready [20] framework to disentangle the oil shocks into their daily constituents of supply, demand and risk shocks. This study, encompassing the period of 2002–2020, covers the three global crises; Global Financial Crisis (GFC), European Sovereign Debt Crisis (ESDC) and Covid-19 pandemic health and economic crisis.

We contribute and extend the extant literature on the interdependence between shocks in crude oil prices, agricultural commodity returns, and volatility by employing a novel approach of shock construction. We use Granger causality tests and employ spillover index, based on the decomposition of variances linked to a multi-variable vector autoregressive methodology. We document many significant findings. First, statistically significant granger causality between oil shocks and agricultural commodities such as grains, live cattle, and wheat. Thus, we can infer that oil shocks can be used to predict the future agricultural commodities can be used to predict future price movement in these agricultural commodities and vice versa. Given both oil and agricultural commodities central position for general population, policy makers can use these results for better forecasting and planning in future. Similarly, investors and portfolio managers, can sue these results for developing optimal portfolio design. Second, we find that, from the point of view of static connectedness, for both, price and volatility spillovers, the livestock is the largest transmitter, while the lean hogs variable behaves as a major receiver. These results are important particularly for designing optimal risk management and hedging strategies. In addition, as both these commodities are part of the food basket, from a regulatory perspective, it can give regulators some insight into designing policies for price control, particularly in developing countries. Third, we show that the dynamic rolling connectedness of the commodities-plus-oil system increases during the periods of financial and economic stresses, global economic crises. This feature makes potentially possible a minimization of forecasting errors under such critical conditions, allowing for a better planning of financial stability recovery and maintenance. This increased connectedness underscores the notion of financialization of commodity markets [40].

Thus our results offer valuable insights, in particular, for policy makers in search of solution for accelerated recoveries from economic downturns and for market players pursuing investment portfolio optimizations. It is especially so for energy and non-energy commodity traders and investors, as they have now a confirmation, that return and volatility spillovers between agricultural commodities and crude oil in both directions have a rather limited expression, while those within the agricultural commodities could be significant. The further investigation in this filed is highly desirable, with one of the possible research directions being an expansion of the analysed commodity-plus-oil system by means of inclusion of other macroeconomic aggregates, such as for instance, equity prices per industry, governmental and corporate debt, foreign exchange investments, Economic policy uncertainty, Geopolitical risks, among other such factors.

## Supporting information

S1 Data (CSV)
